# Residual confounding explains the association between high parity and child mortality

**DOI:** 10.1186/1471-2458-13-S3-S5

**Published:** 2013-09-17

**Authors:** Naoko Kozuki, Emily Sonneveldt, Neff Walker

**Affiliations:** 1 Department of International Health, Johns Hopkins Bloomberg School of Public Health, 615 N. Wolfe St., Baltimore, MD 21205, USA; 2 Futures Institute, 41-A New London Turnpike, Glastonbury, CT 06033 USA

## Abstract

**Background:**

This study used data from recent Demographic and Health Surveys (DHS) to examine the impact of high parity on under-five and neonatal mortality. The analyses used various techniques to attempt eliminating selection issues, including stratification of analyses by mothers’ completed fertility.

**Methods:**

We analyzed DHS datasets from 47 low- and middle-income countries. We only used data from women who were age 35 or older at the time of survey to have a measure of their completed fertility. We ran log-binominal regression by country to calculate relative risk between parity and both under-five and neonatal mortality, controlled for wealth quintile, maternal education, urban versus rural residence, maternal age at first birth, calendar year (to control for possible time trends), and birth interval. We then controlled for maternal background characteristics even further by using mothers’ completed fertility as a proxy measure.

**Results:**

We found a statistically significant association between high parity and child mortality. However, this association is most likely not physiological, and can be largely attributed to the difference in background characteristics of mothers who complete reproduction with high fertility versus low fertility. Children of high completed fertility mothers have statistically significantly increased risk of death compared to children of low completed fertility mothers at every birth order, even after controlling for available confounders (i.e. among children of birth order 1, adjusted RR of under-five mortality 1.58, 95% CI: 1.42, 1.76). There appears to be residual confounders that put children of high completed fertility mothers at higher risk, regardless of birth order. When we examined the association between parity and under-five mortality among mothers with high completed fertility, it remained statistically significant, but negligible in magnitude (i.e. adjusted RR of under-five mortality 1.03, 95% CI: 1.02-1.05).

**Conclusions:**

Our analyses strongly suggest that the observed increased risk of mortality associated with high parity births is not driven by a physiological link between parity and mortality. We found that at each birth order, children born to women who have high fertility at the end of their reproductive period are at significantly higher mortality risk than children of mothers who have low fertility, even after adjusting for available confounders. With each unit increase in birth order, a larger proportion of births at the population level belongs to mothers with these adverse characteristics correlated with high fertility. Hence it appears as if mortality rates go up with increasing parity, but not for physiological reasons.

## Background

Researchers have reported that high parity births have a higher risk of child mortality than lower parity births.[1-5] The question remains whether this association is driven by a causal mechanism or if it is simply a result of selection issues. With each increase in parity, a larger percentage of births belongs to mothers who have characteristics correlated with high fertility. If those characteristics are adverse to child health (e.g. low socioeconomic status, poor quality of or access to health care), we expect the mortality risk to appear higher in high parity births; only mothers with those adverse characteristics contribute high parity births, whereas both mothers with and without those characteristics contribute low parity births.

Studies have tried to disentangle the causal link between high parity and mortality from possible confounding, controlling for variables such as maternal education, wealth, and maternal age.[4,6] Overall, these studies found that controlling for these confounders attenuates the association between parity and mortality, but that a significant association still remains. In our analysis, we explore this issue further by using the mother’s fertility at the end of her reproductive period (completed fertility) as a proxy for confounders, and determine whether controlling for this proxy measure, in addition to available confounders, would account for the association between high parity and under-five/neonatal mortality. We use Demographic and Health Survey (DHS) datasets in our analyses.

The findings will help determine whether a biological link should be included between parity and child mortality in the Lives Saved Tool (*LiST*). *LiST* is a software package that is used to estimate the impact of scaling up interventions on mortality among women and children.[7]* LiST*, through its links to other modules within Spectrum[8], also estimates the impact of family planning on fertility and resulting mortality of women and children. Currently within the model, pregnancies and births decrease as contraceptive prevalence increases or as women switch to more effective contraceptive methods. We will attempt to elucidate the relationship between higher parity and child mortality to see if additional reproductive risk should be taken into account in the model.

## Methods

### Datasets

The analyses used data from DHS, performed in low- and middle-income countries to collect data on a broad range of nationally-representative indicators related to health, including full birth histories of women aged 15-49 living in a household. We identified a total of 49 DHS datasets from Phase V (29 African DHS, 8 Asia, 5 Americas, 7 North Africa/Central Asia/Europe). We limited our analyses to Phase V surveys to have the same control variables available across all datasets. If multiple Phase V surveys were conducted in a single country, we used the most recent survey. We did not include surveys from Sao Tome and Principe and Ukraine due to a low number of overall births for the former and a low number of high fertility mothers for the latter, resulting in 47 countries included in the analyses. The list of countries can be found in Additional file 1 supplemental table 1, along with the included number of births and deaths, stratified by birth order and maternal fertility category.

### Selection of mothers and births

In each country-level dataset, we first selected women who were 35 years of age or older at the time of the survey. Then we used only their births that occurred when they were 18-<35 years old to account for possible confounding by maternal age. We excluded any births that occurred in the five years prior to the mother taking the survey when examining under-five mortality, as we cannot accurately assess survivorship at age five on those children. Similarly, any births that occurred within the month preceding the administration of the survey were excluded for neonatal mortality.

### Data analysis

We first ran log-binominal regression by country to calculate relative risk (RR) between parity and both under-five and neonatal mortality. In our study, parity is defined as the number of live births, with the child of interest labeled by their birth order (i.e. first live birth = parity 1). Parity was examined in two ways. One, parity was made into an ordinal variable, with parity 2-3 as the lowest, parity 4, parity 5, parity 6, and parity ≥7 as the highest, allowing us to examine parity like a continuous variable. We combined parity 2 and 3, as they have been reported in previous literature as having the lowest risk. Primiparous births were excluded from the above analyses, as existing literature suggests that first births have higher risk than parity 2-3. Parity ≥7 were combined because of small sample size. We also examined parity as a categorical variable, comparing parity ≥4 against parity 2-3 as reference. We controlled for wealth quintile, maternal education (none, primary, secondary or above), urban vs. rural residence, maternal age at first birth, calendar year (to control for possible time trends), and birth interval between previous child and index child.

We hypothesized that the association between high parity and child mortality is not due to biological mechanisms, but because as parity increases, a larger proportion of births are represented by children who are born to high risk mothers. We postulated that available confounders are not fully capturing this difference in maternal risk; we therefore took the mothers’ fertility at the time of survey as completed or near-complete fertility (as the woman needed to be age 35 or older at the time of survey to be included in our analyses), and used it as a proxy indicator for residual confounders. (All future references of fertility in this paper refer to the mothers’ completed or near-complete fertility.) We categorized the mothers’ fertility as low or high (low for 1-4 live births, high for ≥5 live births). We ran log-binominal regression by country to calculate RRs of both under-five and neonatal mortality, comparing children of high fertility mothers to children of low fertility mothers. The analysis was stratified by birth order to examine the difference in mortality risk between the two fertility categories, without the impact of parity / birth order. The analysis was run unadjusted and then adjusted for the same confounders listed above. RRs were not available for certain birth orders in some countries due to small sample size. The country-level associations were then meta-analyzed using the metan command in Stata.

Finally, we repeated the first analysis examining the impact of parity on under-five / neonatal mortality, but only among mothers in the high fertility category. We investigated whether the association between high parity and child mortality would still exist when just examining mothers with similar background characteristics. We used log-binominal regression by country to calculate RRs of under-five / neonatal mortality. Unadjusted and adjusted analyses were conducted, with the same control variables listed above, and the associations were meta-analyzed.

We used random effects for the meta-analyses to control for heterogeneity across countries. Standard DHS sample weights were used in the analysis to account for both under- and over-sampling and variation in response rates. The svyset command was used to account for both clustering and stratification in the DHS survey design. Stata Version 12 was used for the analyses.

## Results

### The association between high parity and under-five / neonatal mortality (the “parity effect”)

We report the association between high parity and under-five / neonatal mortality, adjusted for confounders. The result is what the literature often labels as the “parity effect.” For under-five mortality, parity as a continuous variable (excluding primiparity) had an unadjusted RR of 1.08 (95% CI: 1.06, 1.10) and adjusted RR of 1.07 (95% CI: 1.06, 1.09). Parity as a categorical variable (parity ≥4, ref: parity 2-3) had an unadjusted RR of 1.17 (95% CI: 1.13, 1.22) and an adjusted RR of 1.15 (95% CI: 1.11, 1.19). For neonatal mortality, parity as a continuous variable had an unadjusted RR of 1.11 (95% CI: 1.09, 1.13) and an adjusted RR of 1.12 (95% CI: 1.10, 1.15), and parity as a categorical variable had an unadjusted RR of 1.24 (95% CI: 1.17, 1.31) and an adjusted RR of 1.28 (95% CI: 1.21, 1.35).

### Difference in mortality risk, comparing children of high fertility mothers to low fertility mothers

**Figure 1:**
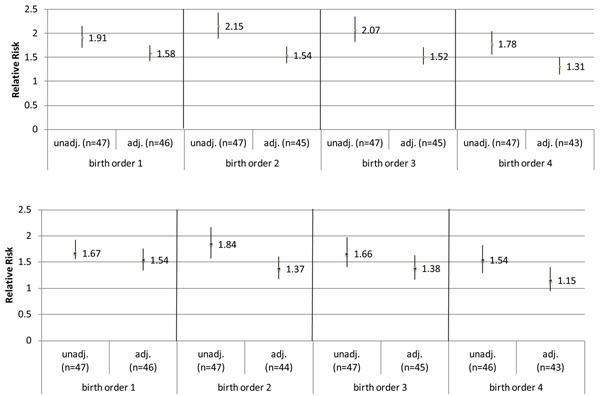
**a: Relative risk of under-five mortality, among children of high completed fertility mothers (reference: low completed fertility), unadjusted vs. adjusted.** *Adjusted RRs are controlled for wealth, maternal education, urban vs. rural residence, calendar year, maternal age at first birth, and birth spacing (for birth order 2-4). **Low completed fertility: 1-4 live births at the end of a mother’s reproductive period, high completed fertility: ≥5 live births at the end of a mother’s reproductive period.

Putting the “parity effect” aside, we calculated the RR of mortality comparing children of high fertility mothers to those of low fertility mothers, stratified by birth order (up to birth order four). The analysis showed that the unadjusted risk of under-five mortality is roughly two times higher in children of high fertility mothers compared to children of low fertility mothers, for each birth order (Figure 1a), with slightly attenuated but still significant associations for neonatal mortality (Figure 1b). The effect size is fairly consistent across birth orders; the effect size for birth order 4 may be more attenuated because mothers with four live births are likely more similar in characteristics to women who belong to the high fertility category. Maternal age should not be a major confounder, as we only included births that occurred when mothers were between ages 18-<35 at time of delivery.

**Figure 2:**
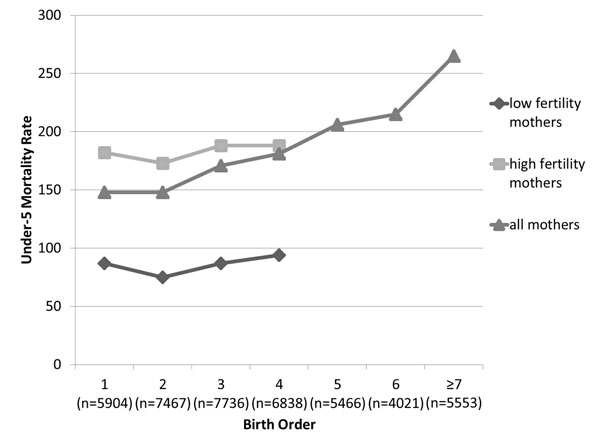
**Under-five mortality rate by birth order for children of low completed fertility mothers, of high completed fertility mothers, and all mothers – example of Nigeria (DHS 2008).** Low completed fertility: 1-4 live births at the end of a mother’s reproductive period, high completed fertility: ≥5 live births at the end of a mother’s reproductive period

**Figure 3:**
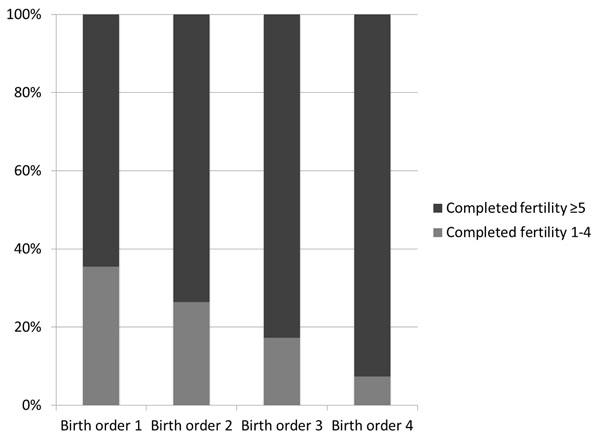
**Composition of birth order 1-4 children, by mother’s fertility category – example of Nigeria (DHS 2008). **Low completed fertility: 1-4 live births at the end of a mother’s reproductive period, high completed fertility: ≥5 live births at the end of a mother’s reproductive period.

We selected several DHS datasets with larger sample sizes (Nigeria in Figure 2, Cambodia and India in Additional file 1 supplemental figures 1a and 1b) to visualize how under-five mortality rates change with birth order for each fertility category. We plotted crude under-five mortality rates at each birth order, both including all children and also stratified by their mothers’ fertility category. We see that at each birth order, children of high fertility mothers have higher mortality risk than children of low fertility mothers. The “all children” line appears to have an increasing trend despite the stratified lines not having increasing trends. This is because the children in the higher risk, high fertility category represent a larger proportion of births as birth order increases; in other words, there are less low risk children “averaging out” the high risk children as birth order increases. (See Figure 3 for proportion of low versus high fertility mothers contributing births to each birth order).

This high versus low fertility gap in child mortality could be driven by differences in various socioeconomic variables. For descriptive purposes, we computed average maternal education level, wealth quintile, and proportion living in rural areas, stratified by whether the mother belonged to the low or high fertility category. We see a consistent trend across countries that low fertility women are better off socioeconomically (higher maternal education and wealth quintile, lower proportion residing in rural areas) (Additional file 1 supplemental table 2). While adjusting for these variables as well as birth interval attenuated the RR of child mortality comparing high fertility to low fertility women, a statistically significant RR remained (Figure 1a and 1b). Control for maternal age at first birth and calendar year of birth did not alter the associations. This suggests that statistically controlling for available socioeconomic and reproductive health variables does not fully account for the confounders correlated with mothers’ completed fertility. Residual confounders leave children of high fertility mothers at greater risk from the very first birth. Thus, examination of the “parity effect” would not be appropriate without acknowledging this residual confounding.

We examined whether the difference in mortality risk comparing children of high fertility and low fertility mothers holds across different geographic regions. The statistically significant difference remained for all four DHS regions for under-five mortality, while some strata lost statistical significance for neonatal mortality (Africa for birth order 2-4, Americas for birth order 4). The magnitude of associations differed across regions, generally with Africa having the lowest effect size and the North Africa/Central Asia/Europe region having the highest (Additional file 1 supplemental table 3a and 3b). The variation in effect size may be due to differences in fertility rates across these regions. High and low fertility women may not differ as much in background characteristics in regions with higher total fertility rate (TFR); high fertility women may be less of outliers in high TFR countries. We extracted TFRs of each country from roughly the midpoint of the maternal birth recall period (see Additional file 1 Supplemental Text 1 for how midpoint was derived). The regional average TFRs were as follows: Africa with 6.36, Asia with 5.07, Americas with 4.98, and North/Africa/Central Asia/Europe with 4.25.

We plotted each country’s log adjusted relative risk of under-five mortality among high fertility mothers (reference: low fertility mothers) for birth order 1, against country-level TFR (Additional file 1 supplemental figure 2) to further explore the possible correlation. There appears to be a linear trend, with log relative risk increasing as country-level TFR decreases. Similar trends were seen in plots for birth order 2, 3, and 4 (not presented). We meta-analyzed the mortality associations comparing children of high fertility and low fertility mothers, stratified by the country-level TFR (TFR 2-4 vs. 4-6 vs. 6-8). The range of TFR was 2.76-7.91. The highest magnitude association was among the countries with TFR 2-4 (RR 2.53, 95% CI 1.89-3.38), and the lowest magnitude among the countries with TFR 6-8 (RR 1.28, 1.16-1.42) for birth order 1. Similar results were seen for birth order 2-4 (Table 1). This may suggest that the gap in child mortality risk between high and low fertility mothers is smaller in high TFR countries.

**Table 1:** Meta-analyzed relative risk of under-five mortality, comparing children of high fertility mothers to low fertility mothers, stratified by country-level total fertility rate

	Birth order 1	Birth order 2	Birth order 3	Birth order 4
TFR 2-<4 (n=7)	2.53 (1.89, 3.38)	2.48 (1.94, 3.17)	2.81 (1.98, 3.99)	1.91 (1.27, 2.86)
TFR 4-<6 (n=14)	1.87 (1.63, 2.14)	1.94 (1.64, 2.29)	1.88 (1.57, 2.26)	1.58 (1.22, 2.06)
TFR 6-<8 (n=22)	1.28 (1.16, 1.42)	1.40 (1.23, 1.59)	1.34 (1.20, 1.49)	1.19 (1.06, 1.35)

TFR = total fertility rate

n represents number of countries that belonged to the specified TFR level

### The association between high parity and under-five/neonatal mortality (the “parity effect”), only among children of high fertility mothers

**Figure 4:**
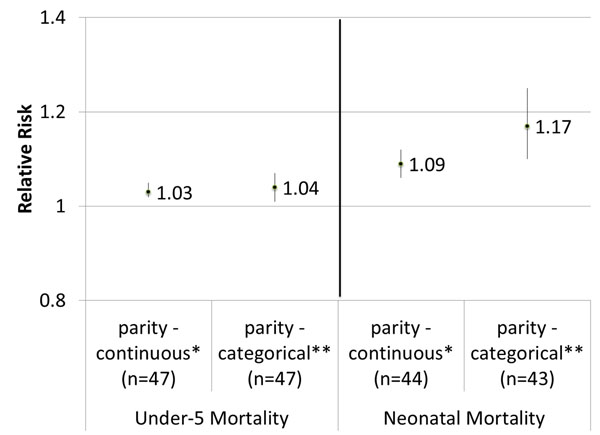
**Adjusted relative risk for under-five / neonatal mortality associated with high parity births, only among children of high completed fertility mothers.** n = number of DHS datasets included *Continuous parity: parity 2-3, 4, 5, 6, ≥7. Primiparous births excluded. **Categorical parity: parity ≥4 (reference: parity 2-3). Primiparous births excluded. Adjusted for wealth, maternal education, urban vs. rural residence, calendar year, maternal age at first birth, and birth spacing (for birth order 2-4).

The residual confounding as illustrated by mothers’ completed fertility does not preclude the existence of a parity effect; we examined the impact of parity on child mortality, only among children of high fertility mothers. There is little evidence to suggest that there is a strong parity effect on child mortality. With parity as a continuous variable, we saw a 3% increased risk in under-five mortality, and as a categorical variable, parity ≥4 saw a 4% increased risk in under-five mortality compared to parity 2-3. Neonatal mortality had slightly higher risk, with 9% increase for parity as a continuous variable and 17% for parity as a categorical variable. (Figure 4) All associations were statistically significant, but attenuated compared to the parity associations calculated for all children (see results from first analysis).

**Figure 5:**
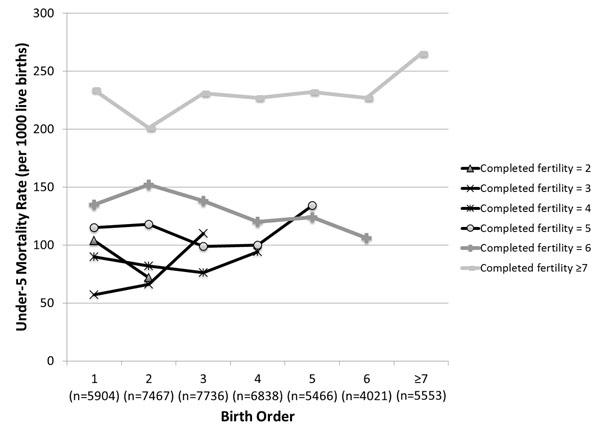
Under-five mortality rate by birth order, stratified by mother’s completed fertility – example of Nigeria (DHS 2008).

The small, but statistically significant “parity effect” among children of high fertility mothers may only exist because of the fertility categorization we chose. We had categorized women with five or more live births as high fertility, but we also expect differences in background characteristics within that category; high fertility as we defined it could range from 5 to as high as 12 or more. To explore this hypothesis, we plotted a figure similar to Figure 2 but with mortality stratified by unit of completed fertility, using the same datasets (Nigeria presented in Figure 5, Cambodia and India presented in Additional file 1 supplemental figures 3a and 3b). We saw a trend of higher completed fertility having higher under-five mortality rates at each birth order, but we did not witness any consistent patterns of mortality rate increasing with birth order among children of the same unit of completed fertility. This suggests that if we repeat the “parity effect” analysis stratified by mothers’ unit of completed fertility, we may no longer see any association between parity and child mortality.

## Conclusions

Previous research has suggested a causal link between high parity and child mortality. However, our analyses indicate that the association is not driven by a physiological effect, but rather maternal characteristics correlated with high completed fertility. Children of high fertility mothers have higher mortality compared to children of low fertility mothers, starting from the very first birth. High parity births appeared to have excess mortality risk in previous literature most likely because as parity increases, a larger proportion of births belongs to these higher risk, high fertility mothers. The statistically significant difference in mortality risk between children of high and low fertility mothers remained even after controlling for available socioeconomic and reproductive health variables. These covariates are similar to those controlled in existing literature, suggesting that previous findings on the parity-child mortality association most likely did not fully account for confounders.

The mortality risk gap between children of high and low fertility women does not preclude the existence of a causal link between high parity and child mortality. However, when we examined the association between parity and child mortality only among children of high fertility mothers, we observed a minimal impact of parity. We defined high fertility here as women with five or more live births, but maternal characteristics correlated with being grand multiparous, compared to being multiparous, may be even more strongly associated with child mortality. If sample size allowed us to examine the parity effect among women in each unit of completed fertility, we would expect the parity associations to attenuate further or even disappear. As our analyses were conducted on cross-sectional data, the findings are inconclusive; a separate paper in this supplement will reexamine the parity effect using prospective cohort data.[9]

Even if a biological association does not exist between parity and child mortality, parity still serves as an important indicator for programs to identify children with high mortality risk. Family planning interventions can reduce child and maternal mortality by limiting the number of births. However, identifying mothers only after they had given birth to many children means that we miss the opportunity to address the equally high mortality risk of their earlier children. The key will be to uncover indicators that are highly correlated with completed fertility and identifiable early in a woman’s reproductive period to mitigate child mortality risk from their very first birth. Many of these factors are already known and prioritized in health programs, such as poverty and low education. However, identification of additional factors could provide previously unseen opportunities. Characteristics like access to and quality of health care may partially explain the residual confounding. These characteristics may drive both higher child mortality risks and low access to family planning among high fertility mothers. In DHS datasets, data on careseeking is only available for a mother’s most recent birth, so we were unable to further explore this hypothesis. There may also be environmental factors like sanitation and pollution that is highly correlated with low socioeconomic status that may not be fully captured by available variables.

Similar analytical methods as those used here were found in studies conducted in a Jerusalem hospital setting. They examined the birth history of only women who had high fertility (fertility 7-10), and saw no significant differences in low birthweight prevalence across all of their births.[10] When grand-multiparous women were stratified by socioeconomic status, they witnessed no effect of parity on child outcomes.[11] These studies were, however, small in sample size and were conducted in a developed country setting. There have also been other studies showing that grandmultiparous women who were economically stable with good access to care saw no increased risk in child mortality outcomes[12-14]. Another study that utilized World Fertility Surveys also suggested weak impact of high order births, and suggested that birth intervals may be the driving confounder that has previously made high parity appear to be high risk.[15] Some have also argued that high parity makes an impact on child survival only when interacting with short birth intervals.[16]

As our analyses raised sufficient doubts concerning the biological relationship between high parity and child mortality, we do not recommend an association to be included in the *LiST* model. Mothers with high completed fertility appear to have characteristics adverse to child health, and this heightened risk holds across all of their births, regardless of birth order. At the population level, a larger proportion of births are represented by these high risk children as birth order goes up; hence it appears as if there is a biological association between high parity and child mortality risk.

## Competing interests

The authors declare that they have no competing interests. The publications costs for this paper are from a grant from the Bill & Melinda Gates Foundation to the US Fund for UNICEF (grant 43386 to "Promote evidence-based decision making in designing maternal, neonatal, and child health interventions in low- and middle-income countries”).

## Author’s contributions

NK conducted the statistical analyses, NK and NW both worked on all remaining aspects of the work for the manuscript, and ES contributed to the study design and reviewed the manuscript.

## Supplementary Material

Additional file 1 **Supplemental figures, tables, and text.** The file is a word document that contains supplemental figures, tables, and text. Click here for file

## Declarations

The publication costs for this supplement were funded by a grant from the Bill & Melinda Gates Foundation to the US Fund for UNICEF (grant 43386 to "Promote evidence-based decision making in designing maternal, neonatal, and child health interventions in low- and middle-income countries”). The Supplement Editor is the principle investigator and lead in the development of the Lives Saved Tool (LiST), supported by grant 43386. He declares that he has no competing interests.

This article has been published as part of *BMC Public Health* Volume 13 Supplement 3, 2013: The Lives Saved Tool in 2013: new capabilities and applications. The full contents of the supplement are available online at http://www.biomedcentral.com/bmcpublichealth/supplements/13/S3.
