# Supplementary material for: Residual confounding explains the association between high parity and child mortality
Source: BMC Public Health. 2013 Sep 17;13(Suppl 3):S5. doi: 10.1186/1471-2458-13-S3-S5 (PMC3847621; doi:10.1186/1471-2458-13-S3-S5)
Supplement: Additional file 1 — Supplemental figures, tables, and text. The file is a word document that contains supplemental figures, tables, and text. [file 1471-2458-13-S3-S5-S1.pdf]

Kozuki N, Sonneveldt E, and Walker N. Residual confounding explains the association between high parity and child mortality.

Supplemental Figure 1a: Under-5 mortality rate by birth order for children of low completed fertility mothers, of high completed fertility mothers, and all mothers – Example of Cambodia (DHS 2010)

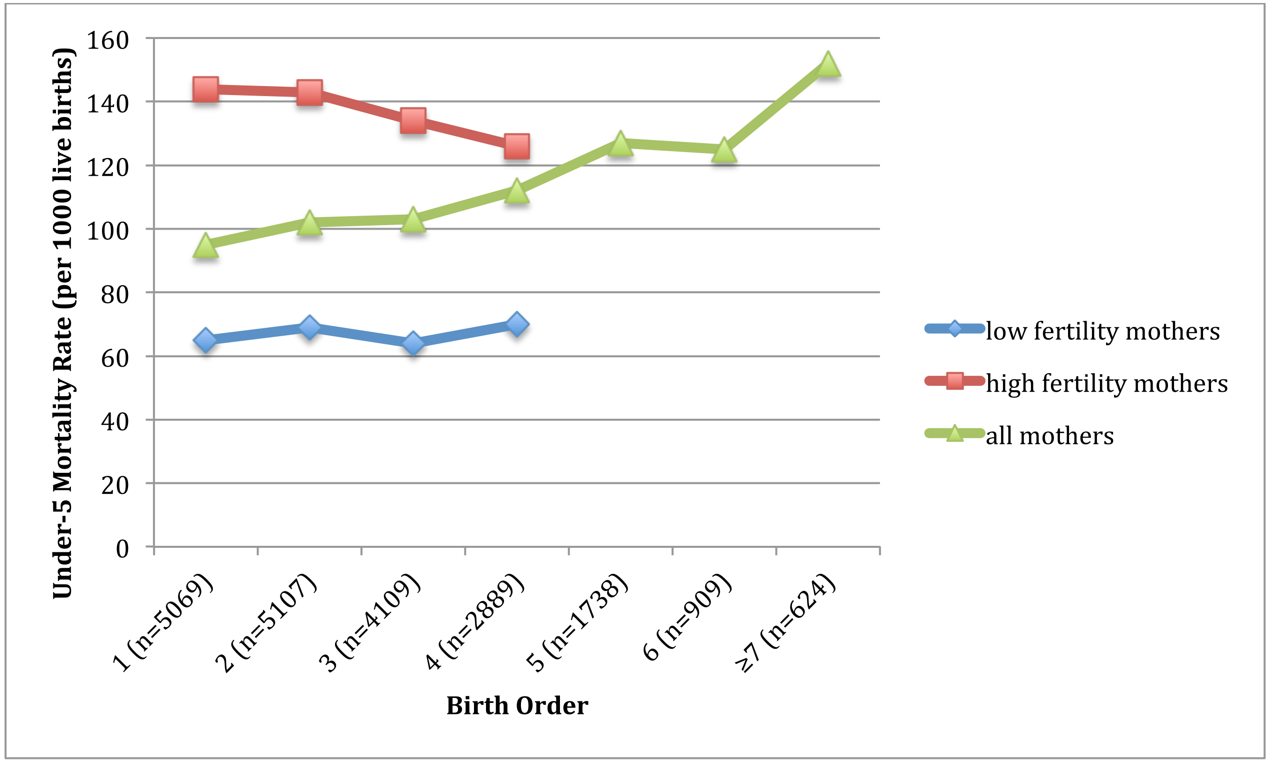

Low completed fertility: 1-4 live births at the end of a mother's reproductive period, high completed fertility: ≥5 live births at the end of a mother's reproductive period

Supplemental Figure 1b: Under-5 mortality rate by birth order for children of low completed fertility mothers, of high completed fertility mothers, and all mothers – Example of India (DHS 2005-06)

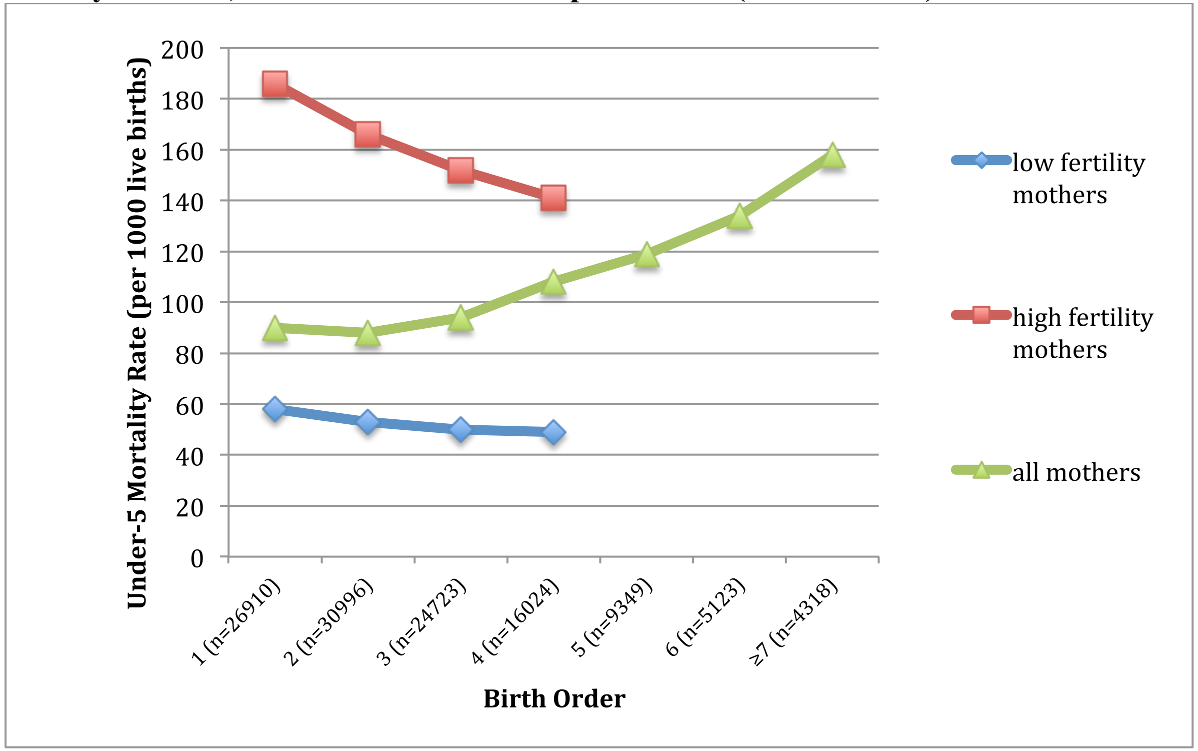

Low completed fertility: 1-4 live births at the end of a mother's reproductive period, high completed fertility: ≥5 live births at the end of a mother's reproductive period

Supplemental Figure 2: Country-level log-adjusted relative risk of under-5 mortality among high completed fertility mothers (reference: low completed fertility mothers), plotted against country TFR, only among birth order 1

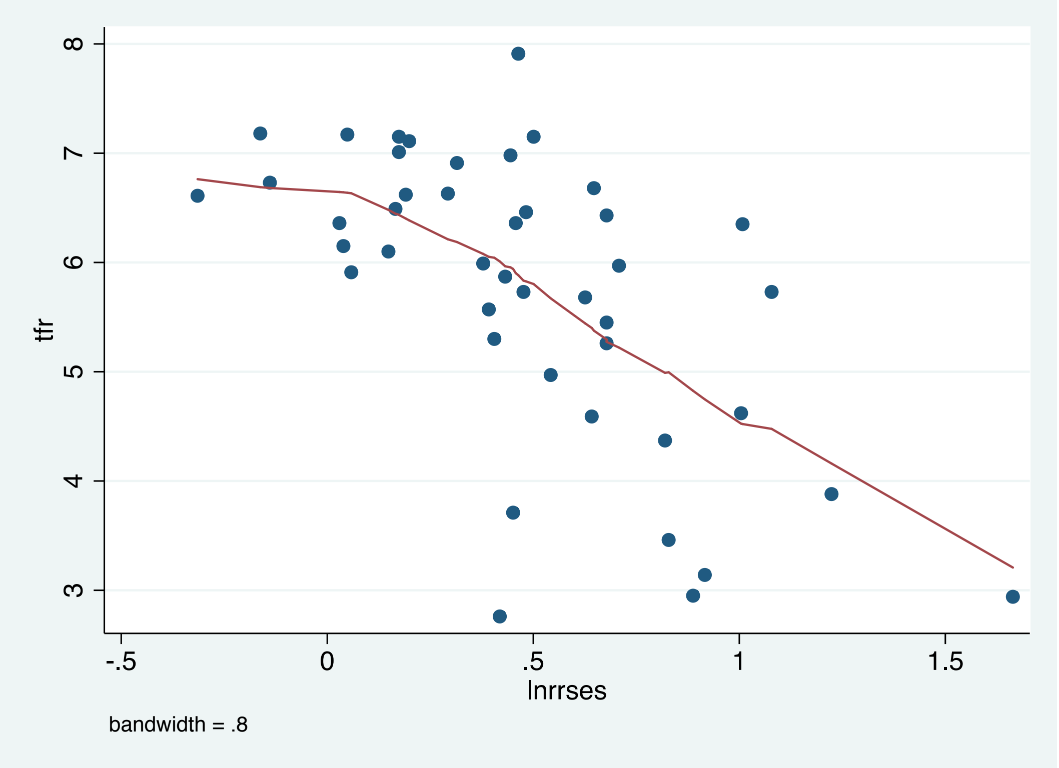

47 DHS countries included

Supplemental Figure 3a: Under-5 mortality rate by birth order, stratified by mother's completed fertility – Example of Cambodia (DHS 2010)

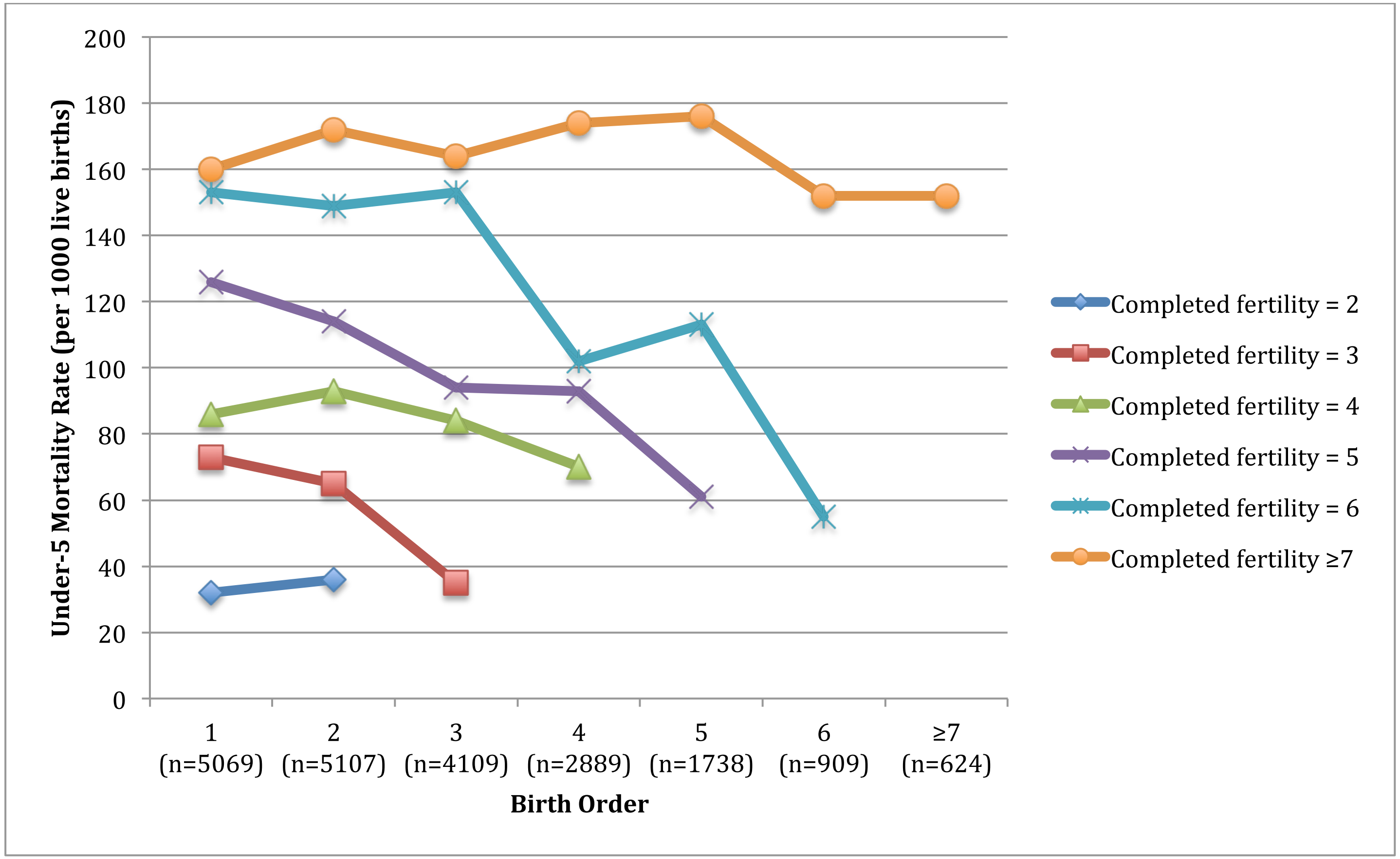

Completed fertility: number of births at the end of mothers' reproductive period

Supplemental Figure 3b: Under-5 mortality rate by birth order, stratified by mother's completed fertility – Example of India (DHS 2005-06)

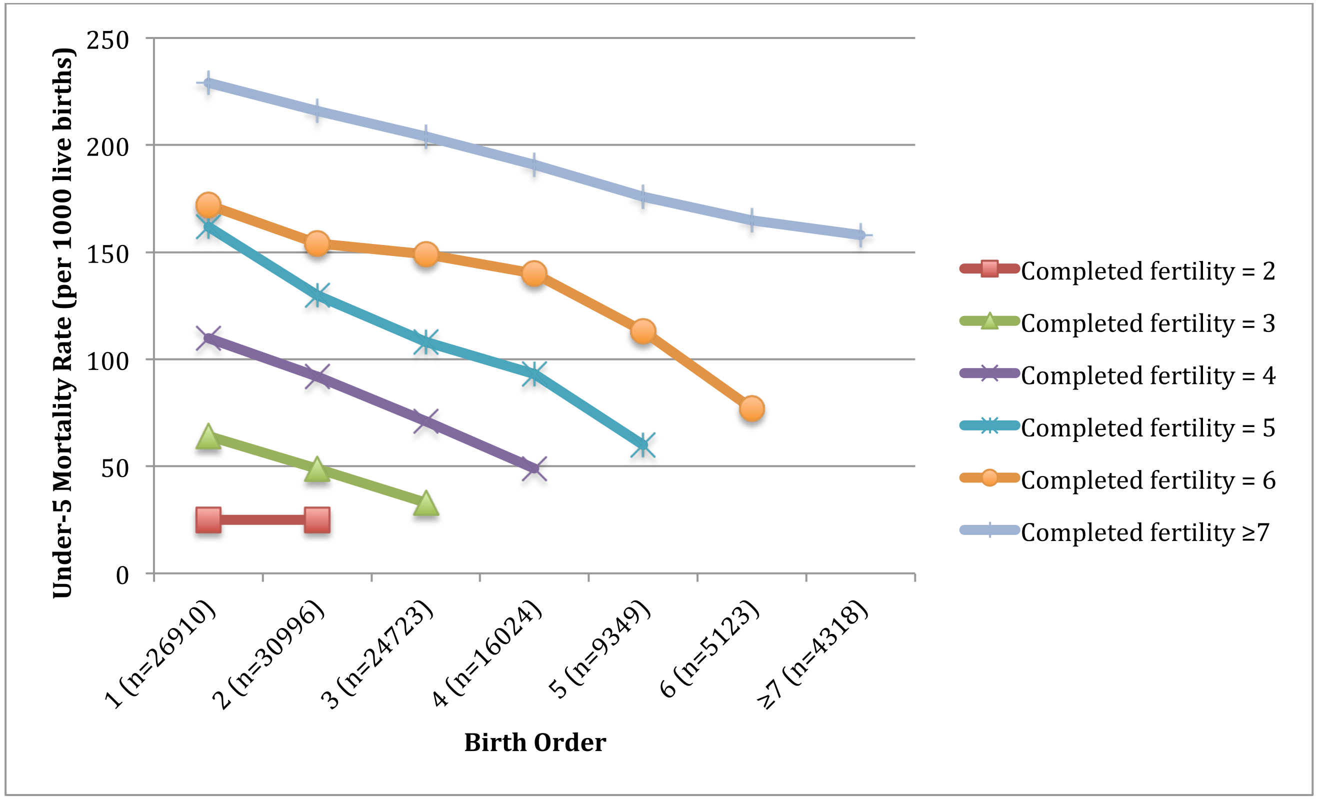

Completed fertility: number of births at the end of mothers' reproductive period

Supplemental Table 1: DHS country-specific number of births and deaths included in the analyses

Country	total births	total deaths	low fertility %	high fertility %	Birth order 1				Birth order 2				Birth order 3				Birth order 4				Birth order 5+	
					low fertility		high fertility		low fertility		high fertility		low fertility		high fertility		low fertility		high fertility		high fertility	
					Birth	Death	Birth	Death	Birth	Death	Birth	Death	Birth	Death	Birth	Death	Birth	Death	Birth	Death	Birth	Death
**Africa**																						
Benin	20652	2581.50	11.56	88.44	882	80.61	1850	344.10	797	66.31	2522	407.30	534	43.25	3504	408.57	207	20.29	3432	400.86	6924	820.49
Burkina Faso	18390	3689.03	11.85	88.15	619	95.51	2075	479.12	595	55.10	2855	604.12	420	48.89	3020	575.91	156	20.76	2936	585.73	5714	1231.94
Cameroon	11596	1572.42	14.22	85.78	545	61.20	1004	131.02	559	52.32	1528	192.38	391	28.11	1713	239.31	163	20.03	1716	237.49	3977	609.28
Chad	8423	1702.29	6.92	93.08	194	37.38	690	124.27	205	37.25	1084	177.56	169	33.04	1271	241.24	77	11.72	1284	276.70	3449	762.92
Congo-Brazzaville	6210	671.30	25.77	74.23	554	43.49	490	51.45	565	46.22	836	97.39	368	27.89	923	110.21	143	9.58	887	89.76	1444	194.51
DRC	11183	1715.47	11.97	88.03	509	47.29	1169	174.41	483	40.14	1654	244.13	300	22.86	1767	269.47	119	18.69	1692	261.58	3490	630.64
Ethiopia	16588	2848.16	9.64	90.37	756	99.72	1417	245.28	760	110.73	2195	409.81	506	57.79	2567	478.23	231	24.81	2545	467.77	5611	925.25
Ghana	5494	631.81	31.07	68.93	538	42.02	521	86.59	541	42.09	735	116.50	358	21.44	797	96.20	115	7.49	740	72.08	1149	156.49
Guinea	12147	2818.10	12.59	87.41	523	97.23	1194	329.90	520	56.52	1782	414.14	372	49.22	2002	478.08	152	37.39	1927	448.41	3675	904.79
Guyana	5423	256.51	57.51	42.49	989	54.59	316	21.14	924	25.41	484	22.80	581	21.96	533	38.27	190	5.76	527	24.35	879	49.75
Kenya	9168	796.70	25.18	74.82	788	34.04	798	56.42	754	21.34	1151	105.43	497	36.38	1272	117.28	185	10.92	1254	122.52	2469	294.06
Lesotho	6376	466.09	48.94	51.06	1049	67.35	574	58.49	967	55.99	715	63.28	613	35.43	739	45.82	214	9.35	693	63.27	812	74.70
Liberia	8817	1858.62	18.4	81.6	518	50.30	668	159.12	552	72.64	1143	257.29	366	47.07	1361	327.46	133	13.62	1321	279.92	2755	657.62
Madagascar	19800	2231.46	23.58	76.42	1767	110.08	1836	233.36	1642	127.91	2475	321.01	1046	68.41	2679	341.84	371	23.60	2584	291.48	696	90.97
Malawi	26702	4803.69	12.09	87.91	982	137.48	2658	537.45	1079	165.09	3905	748.59	795	81.33	4318	783.29	367	38.28	4261	741.84	8337	1571.52
Mali	20644	4979.33	9	91	666	138.59	1971	539.46	610	65.27	2744	678.32	425	82.32	3109	757.04	147	15.99	3119	730.47	7853	1975.03
Mozambique	13925	2881.08	14.98	85.02	732	121.73	1275	275.53	85	8.87	363	75.21	510	64.11	2118	473.37	192	25.48	2095	459.64	4414	992.71
Namibia	8439	567.10	42.37	57.63	1299	47.93	735	53.14	1176	74.44	967	67.01	697	46.21	1013	72.73	226	16.79	946	68.40	1380	124.61
Niger	13533	3494.22	5.78	94.22	314	55.39	1100	306.13	319	56.11	1778	482.90	216	50.41	2116	519.05	84	13.02	2107	599.44	5499	1402.79
Nigeria	42985	7965.12	13.89	86.11	1947	168.42	3957	718.20	1822	137.38	1822	315.57	5645	490.55	6513	1224.44	460	43.42	6378	1197.15	15040	3460.70
Rwanda	13876	2479.64	13.44	86.56	741	89.22	1998	343.06	628	93.45	2214	384.57	383	69.48	2249	409.99	132	21.69	2100	382.41	3451	684.14

Senegal	16649	2344.18	13.48	86.52	780	74.02	1688	255.73	711	64.06	2344	303.55	446	38.31	2607	397.57	153	17.12	2532	353.72	5388	849.15
Sierra Leone	7823	1509.06	22.55	77.45	689	61.11	628	149.53	665	66.43	956	204.01	437	37.28	1127	231.82	147	19.39	1106	231.26	2068	485.98
Swaziland	4880	324.52	26.09	73.91	409	22.90	362	29.58	432	20.56	586	49.11	291	12.77	671	48.85	114	8.70	671	44.62	1344	88.17
Tanzania	12968	1685.84	18.35	81.65	85	11.24	195	28.82	75	8.07	237	32.75	516	58.62	2011	269.07	176	14.17	1952	256.30	3738	471.36
Uganda	11320	1933.46	7.82	92.18	41	5.92	170	28.12	40	5.90	290	50.87	16	1.06	317	56.68	13	1.98	314	54.20	767	134.38
Zambia	7629	1128.33	12.4	87.6	55	9.05	95	13.99	41	4.76	171	28.30	27	3.71	197	33.27	11	1.19	156	19.80	2477	357.18
Zimbabwe	7458	458.67	32.33	67.67	837	39.59	688	52.63	838	33.35	977	59.11	593	19.33	1057	88.26	216	11.51	1027	72.20	1716	114.97
**Asia**																						
Bangladesh	12604	1566.68	40.14	59.86	1274	155.56	567	131.60	1769	146.65	1188	227.50	1346	89.24	1506	261.44	622	25.07	1528	212.85	2804	325.26
Cambodia	20445	2173.30	42.11	57.89	3137	202.96	1932	278.40	2851	197.57	2526	362.23	1821	116.18	2288	307.51	712	49.98	2177	273.65	3271	429.16
India	117443	11732.56	51.48	48.52	20715	1207.68	6195	1149.79	22011	1170.99	8985	1487.02	14548	721.58	10175	1550.67	5833	282.90	10191	1432.85	18790	2474.64
Indonesia	41898	3247.10	61.16	38.85	8885	430.92	2403	337.14	8533	377.16	3253	464.53	4736	194.18	3641	405.24	1478	89.71	3514	400.60	5455	710.79
Maldives	11052	664.23	32.85	67.15	1057	52.22	717	75.50	1058	39.99	1256	110.03	735	28.89	1455	107.23	306	6.61	1478	97.55	2990	156.98
Nepal	12394	1689.30	33.87	66.13	1259	104.62	1119	228.16	1367	60.56	1462	325.88	1053	46.33	1535	277.99	455	28.35	1510	241.15	2634	385.35
Pakistan	18756	1845.59	16.85	83.15	1020	71.09	2080	273.94	938	48.78	2520	279.22	694	28.80	2666	285.00	314	14.41	2605	237.84	5919	616.76
Philippines	14722	715.49	43.81	56.19	2410	68.93	1297	87.29	2039	49.55	1547	111.07	1247	25.69	1596	101.67	393	12.85	1520	93.63	2673	178.56
**Americas**																						
Bolivia	19600	2352.00	31.48	68.52	2396	148.79	1652	288.11	2080	118.77	2208	356.15	1267	60.82	2387	376.19	456	19.93	2341	354.43	4813	620.88
Colombia	45148	1413.13	71.42	28.58	11330	234.53	1859	117.86	10145	249.57	2825	156.22	5613	107.21	3187	185.48	1741	49.10	3141	156.11	5307	262.70
Dominican Republic	27228	1326.00	65.83	34.17	5310	169.39	1114	79.87	5486	192.56	1763	165.90	4010	131.53	2065	145.38	1435	53.67	2098	129.66	3947	335.89
Haiti	11951	1668.36	21.32	78.68	952	115.29	1325	195.31	834	60.55	1637	252.26	497	41.10	1701	239.67	188	26.81	1622	212.64	3195	529.41
Honduras	22659	15396.79	94.34	5.66	2161	72.83	1800	144.72	2055	56.72	2580	194.02	1421	39.93	2853	213.12	571	11.42	2830	194.70	6388	395.42
**Other**																						
Albania	8841	354.52	84.25	15.75	2857	86.00	315	39.69	2620	55.81	333	31.60	1327	37.16	333	48.48	325	16.90	317	36.52	414	28.44
Armenia	6553	361.73	91.25	8.75	2315	93.29	112	26.92	2205	87.98	116	26.76	1193	59.05	115	17.86	261	148.51	113	27.06	123	9.73
Azerbaijan	8467	684.98	80.53	19.47	2617	153.88	327	75.90	2344	105.95	335	77.72	1345	63.89	338	90.11	385	17.17	333	74.73	443	46.07
Egypt	24964	1575.23	48.1	51.9	3778	122.79	1868	230.14	3645	69.98	2383	291.92	2578	47.95	2603	268.89	912	17.78	2564	205.38	4633	401.22

Jordan	22895	595.27	20.85	79.15	1311	17.96	2757	105.32	1174	5.87	3219	117.49	823	8.89	3329	109.19	343	0.96	3185	72.62	6754	176.95
Moldova	6205	248.82	89.2	20.8	2644	92.54	114	11.14	2112	67.80	124	17.77	690	20.08	125	13.53	147	3.68	120	8.76	129	10.07
TOTAL	82292<br>3	110004.<br>77																				

* The decimals in the death columns are result of tabulation using survey weights.

**Low completed fertility: 1-4 live births at the end of mothers' reproductive period, high completed fertility: ≥5 live births at the end of mothers' reproductive period

Supplemental Table 2: DHS summary statistics of socioeconomic indicators, by country

	maternal education (mean, category 0 - no education, 1 - primary, 2 - secondary, 3 - higher)		wealth quintile (mean, 1 - poorest, 5 - richest)		rural residence (%)	
country	low fertility	high fertility	low fertility	high fertility	low fertility	high fertility
**Africa**						
Benin	0.71	0.19	3.62	2.71	45.24	67.98
Burkina Faso	0.38	0.07	3.52	2.83	65.84	87.42
Cameroon	1.22	0.83	3.46	2.79	36.43	55.85
Chad	0.22	0.18	3.01	2.83	74.83	82.16
Congo-Brazzaville	1.76	1.32	3.54	2.69	30.79	59.06
DRC	1.19	1	3.13	2.84	53.84	62.1
Ethiopia	0.49	0.12	3.41	2.92	68.64	90.88
Ghana	1.51	0.83	3.48	2.56	42.61	69.48
Guinea	0.34	0.15	3.13	2.71	63.09	80.27
Guyana	1.78	1.65	3.46	2.37	66.86	77.83
Kenya	1.57	0.99	3.83	2.59	64.9	91.39
Lesotho	1.58	1.17	3.75	2.68	58.54	86.35
Liberia	0.88	0.48	3.32	2.73	49.74	72.81
Madagascar	1.4	0.97	3.71	2.79	72.85	91.57
Malawi	1.03	0.67	3.47	2.9	76.4	87.4
Mali	0.4	0.17	3.37	2.79	57.28	75.34
Mozambique	0.64	0.48	3.17	2.81	60.8	68.98
Namibia	1.7	1.09	3.56	2.58	43.25	68.75
Niger	0.27	0.12	3.12	2.94	74.17	82.96
Nigeria	1.37	0.69	3.54	2.74	53.33	72.55
Rwanda	0.82	0.6	2.89	2.79	79.95	87.8

Senegal	0.65	0.26	3.63	2.85	38.38	60.21
Sierra Leone	0.57	0.26	3.13	2.85	63.57	72.77
Swaziland	1.75	1.17	3.71	2.73	61.11	85.08
Tanzania	0.97	0.73	3.39	2.67	63.3	85.02
Uganda	1.06	0.7	3.44	2.88	78.51	90.11
Zambia	1.55	1.02	3.66	2.85	45.97	67.82
Zimbabwe	1.58	0.99	3.67	2.6	47.79	80.62
**Asia**						
Bangladesh	0.87	0.49	3.31	2.83	71.86	81.77
Cambodia	1.06	0.78	3.33	2.64	76.21	90.5
India	1.11	0.38	3.64	2.66	59.68	78.1
Indonesia	1.39	1.02	3.28	2.56	54.45	64.55
Maldives	0.98	0.35	3.43	2.56	53.27	82.17
Nepal	0.39	0.1	3.36	2.59	80.01	92.08
Pakistan	0.78	0.31	3.47	2.93	57.08	68
Philippines	2.14	1.56	3.49	2.41	40.99	61.53
**Americas**						
Bolivia	1.75	0.98	3.77	2.44	22.26	55.53
Colombia	1.85	1.15	3.35	2.08	18.15	43.08
Dominican Republic	1.69	1.03	3.45	2.32	25.55	40.52
Haiti	0.99	0.39	3.58	2.45	48.3	74.51
Honduras	1.46	0.89	3.83	2.52	30.1	63.78
**Other**						
Albania	1.58	1.16	3.11	1.97	53.69	82.09
Armenia	2.19	2.03	3.07	2.15	36.33	65.44
Azerbaijan	2.13	1.88	3.24	2.36	38.73	63.69
Egypt	1.4	0.53	3.5	2.38	43.77	72.12
Jordan	2.27	1.94	3.49	3.13	10.47	17.44

Moldova	2.17	1.99	3.22	1.87	57.82	86.91
**AVERAGE**	**1.23**	**0.81**	**3.43**	**2.64**	**53.55**	**73.28**

Low completed fertility: 1-4 live births at the end of mothers' reproductive period, high completed fertility: ≥5 live births at the end of mothers' reproductive period

Supplemental Table 3a: Meta-analyzed adjusted relative risk of under-5 mortality among children of high completed fertility mothers (reference: low completed fertility), stratified by birth order and geographic region

	Africa		Asia		Americas		Other	
	N	aRR (95% CI)	N	aRR (95% CI)	N	aRR (95% CI)	N	aRR (95% CI)
Birth order 1	28	1.33 (1.20, 1.48)	8	1.93 (1.69, 2.21)	5	1.73 (1.27, 2.37)	5	3.27 (2.44, 4.39)
Birth order 2	28	1.28 (1.15, 1.42)	8	1.92 (1.63, 2.27)	5	1.74 (1.50, 2.03)	4	3.54 (2.74, 4.58)
Birth order 3	27	1.21 (1.10, 1.34)	8	2.01 (1.76, 2.30)	5	1.92 (1.63, 2.27)	5	3.43 (2.61, 4.51)
Birth order 4	27	1.10 (0.99, 1.21)	8	1.77 (1.42, 2.20)	5	3.54 (2.74, 4.58)	3	2.41 (1.57, 3.70)

Low completed fertility: 1-4 live births at the end of mothers' reproductive period, high completed fertility: ≥5 live births at the end of mothers' reproductive period
N = Number of countries included in the analysis

Supplemental Table 3b: Meta-analyzed adjusted relative risk of neonatal mortality among children of high completed fertility mothers (reference: low completed fertility), stratified by birth order and geographic region

	Africa		Asia		Americas		Other	
	N	aRR (95% CI)	N	aRR (95% CI)	N	aRR (95% CI)	N	aRR (95% CI)
Birth order 1	28	1.21 (1.05, 1.39)	8	2.02 (1.81, 2.27)	5	1.81 (1.35, 2.42)	5	3.28 (2.41, 4.45)
Birth order 2	28	1.03 (0.87, 1.22)	8	2.00 (1.77, 2.27)	5	1.66 (1.22, 2.26)	3	4.62 (2.88, 7.43)
Birth order 3	28	1.02 (0.86, 1.20)	8	1.94 (1.55, 2.43)	5	1.76 (1.07, 2.90)	4	2.71 (1.81, 4.06)
Birth order 4	28	0.99 (0.82, 1.18)	7	1.56 (1.05, 2.32)	5	1.19 (0.55, 2.56)	3	1.97 (1.13, 3.43)

Low completed fertility: 1-4 live births at the end of mothers' reproductive period, high completed fertility: ≥5 live births at the end of mothers' reproductive period

Supplemental Text 1

Based on the exclusion criteria we instituted, the earliest included births should have occurred 31 years prior to the survey, or when a woman surveyed at age 49 (oldest age of eligibility for reproductive health-related questioning on DHS) would have given birth at age 18, and the last births should have occurred 5 years prior to the survey. This would mean that the midpoint of the recall period would be the midpoint between 31 years and 5 years prior to the survey, or 18 years prior to the survey.
